# A lightweight multi-branch transformer architecture for Kazakh Sign Language recognition under signer-independent conditions

**DOI:** 10.3389/frai.2026.1947364

**Published:** 2026-10-27

**Authors:** Nurzada Amangeldy, Aigerim Yerimbetova, Dusmat Zhamangarin, Ulmeken Berzhanova, Bekbolat Kurmetbek, Nazerke Gazizova

**Affiliations:** 1 Institute of Information and Computational Technologies of the Committee of Science of the Ministry of Science and Higher Education of the Republic of Kazakhstan, Almaty, Kazakhstan; 2 Faculty of Information Technologies, L.N. Gumilyov Eurasian National University, Astana, Kazakhstan; 3 SignBridge LLP, Astana, Kazakhstan; 4 School of Engineering and Information Technology, META University, Almaty, Kazakhstan; 5 Faculty of Information Technology and Artificial Intelligence, Farabi University, Almaty, Kazakhstan

**Keywords:** deep learning, Kazakh Sign Language, landmark-based recognition, MediaPipe Holistic, sign language recognition, signer-independent evaluation, transformer

## Abstract

Sign Language Recognition (SLR) in low-resource sign languages is still limited due to the lack of annotated data, high variability of signers, and lack of benchmarks; Kazakh Sign Language (KzSL) is a representative example of such a low-resource language. The proposed research introduces a novel method for isolated KzSL recognition based on a purpose-collected dataset of RGB video streams of five signers, represented by 1662-dimensional per-frame pose, facial, and hand landmark vectors extracted using MediaPipe Holistic. The final dataset consists of 39,979 gesture samples across 187 output classes (186 gesture classes and 1 no_event class), each of which is resampled into a temporal landmark tensor 56 × 1662. A lightweight model, Transformer model (SignTransformerModel, 3,529,272 trainable parameters), is proposed with two parts: (1) Each landmark is projected through a separate branch of a MultiBranchStem, a branch representation is obtained for each landmark and fused into a unified representation of 256 dimension; (2) the fused sequence is processed by a four-layer Transformer encoder with eight attention heads, aggregated into a single sequence representation by a TemporalAttentionPool, which combines attention and max pooling, and then passed to a classification layer. The model was compared against three baseline architectures—a Standard Transformer, a Temporal Convolutional Network, and a Bidirectional GRU—and obtained a mean accuracy of 0.6698 ± 0.0376, a mean macro F1 of 0.4207 ± 0.0420, and a mean weighted F1 of 0.6546 ± 0.0375 over five outer test partitions of a nested, signer-independent Leave-One-Signer-Out cross-validation, outperforming all three baselines in macro F1 on every partition. The results show that compact landmark-based Transformer models are feasible for the recognition of KzSL, though aggregate accuracy is substantially inflated by the dominant no_event class (gesture-only accuracy: 0.4490), and that inter-signer variability and incomplete class coverage by signers as well as recurrent class confusions exist and present clear directions for expanding the dataset and fine-tuning the model with expert guidance.

## 1 Introduction

SLR is a challenging task of multiple disciplines that requires computer vision, sequence processing, and linguistic modeling. The development of computational models for SLR over the period from 2015 to 2025 demonstrates a clear transition from classical algorithms to deep learning (Al Abdullah et al., 2024). Early SLR systems were primarily based on the Hidden Markov Model (HMM) (Yang et al., 2017), the Support Vector Machine (SVM) (Amangeldy et al., 2022; Olszewska and Quinn, 2019), and K-Nearest Neighbors (KNN), which were employed to classify handcrafted features and to statistically model sign sequences (Al Abdullah et al., 2024; Tubaiz et al., 2015). Despite the development of deep learning, classical methods such as HMM, SVM, and KNN continue to be used owing to their low computational requirements, their stability when working with the small datasets typical of low-resource sign languages, and the higher interpretability of such models compared with deep neural network architectures.

Among contemporary deep learning architectures, convolutional neural networks (CNNs) are considered the most widely used architecture for extracting spatial features such as hand shape, position, and orientation (Al Khuzayem et al., 2024; Amangeldy et al., 2023a; Gao et al., 2025). CNNs have proven effective when working with multimodal data such as RGB-D (color and depth), skeletal data, and hand keypoints. Owing to the mechanisms of sparse connectivity and parameter sharing, CNNs provide high statistical efficiency and make it possible to identify local spatial features of human movements more accurately when processing gesture data.

To extract spatiotemporal features from video sequences simultaneously, three-dimensional convolutional neural networks (3D CNNs) are actively used, since they can take into account both the spatial structure of a frame and the dynamics of motion over time (De Castro et al., 2023). Unlike classical CNNs, which operate only on two-dimensional images, 3D CNNs generalize convolution to three-dimensional tensors, where the additional dimension corresponds to time or to the depth of the video stream. This allows the network to learn filters that respond not only to the shape and position of the hands, but also to characteristic movement trajectories, changes in posture, and the ordering of gestures over time. Due to the joint modeling of spatial and temporal features, 3D CNNs are highly effective in recognizing dynamic gestures and continuous sign language, where the relationships between consecutive frames of a video stream are critical (Gionfrida et al., 2022).

Recurrent neural networks (RNNs) and their advanced modifications—Long Short-Term Memory (LSTM) and the Gated Recurrent Unit (GRU)—are widely used to model dynamic gestures, where it is critical to take into account the temporal dependencies between video stream frames (Rastgoo et al., 2024; Bahuguna et al., 2025). These architectures efficiently process data sequences and make it possible to recognize not only individual gestures but also entire sentences in continuous sign language (Amangeldy et al., 2023b; Wang et al., 2025). The LSTM and GRU models are distinct from classical RNNs because they address the vanishing gradient problem with a mechanism of retaining or forgetting information over time via a series of gates. Bidirectional LSTM (BiLSTM) networks are also commonly employed to enhance the accuracy of sequence analysis, being able to consider context from the previous and following frames of the video stream to recognize complex video-sequence structures and context-sensitive movements (Bahuguna et al., 2025).

Deep learning–based generative approaches, such as Generative Adversarial Networks (GANs) and Variational Autoencoders (VAEs), are in use in the SLR tasks to generate and augment data, particularly when the number of labeled training samples is limited (Elakkiya et al., 2021). GANs are a pair of neural networks: a generator network, which learns to generate realistic gestural data; and a discriminator network, which learns to tell the synthetic data apart from the real. Adversarial process allows the generation of high-quality synthetic samples that enhance the models' robustness against gestures variations, inter-signer differences, and video stream noise. In turn, VAEs learn compact latent representations of gesture data, enabling them to be reconstructed and new examples to be explored from the learnt space of latent features. The employment of GANs and VAEs can enhance the quality of continuous SLR, generalize models, and decrease overfitting in low-resource situations.

As well as generative methods, a specific trend of ongoing research is based on skeletal and keypoint-based representations of gestures and the processing of these representations using graph convolutional networks (GCNs) (Meng and Li, 2021). Within these models, the human body is considered a graph, with nodes representing the joints and body key points, and the edges representing connections in terms of anatomy and spatiotemporal interactions between these nodes. In graph convolution, the features of each node are updated by aggregating information from neighboring nodes, allowing for the ability to effectively model the relationship between hand, head, and body movements and facial expressions. Unlike traditional CNNs, GCNs are able to directly incorporate the topological structure of the human skeleton and to analyze the spatiotemporal dynamics of gestures more accurately. Consequently, GCNs are very useful for identifying sign sequences with a dynamic or continuous nature, particularly when paired with temporal modules and attention mechanisms.

The further development of SLR systems has led to the emergence of hybrid and combined architectures that unite the advantages of different deep learning models. The most common are CNN-LSTM (Rastgoo et al., 2021), ResNet-BiLSTM, LSTM-GRU, Transformer (Tan et al., 2023; Zhang and Kong, 2024), and attention-based (Zhang et al., 2018) architectures (Ismail et al., 2022), in which convolutional networks are responsible for extracting the spatial features of a frame (Amin et al., 2021; Kothadiya et al., 2022), while recurrent or temporal modules model the sequence of movements over time. A recently popular class of transformer models using self-attention mechanisms that can adaptively identify the importance of the various parts of the input sequence (Balaji and Ranjan Prusty, 2024) is particularly prevalent. This is very important for continuous SLR, where important information could be found in very distant parts of the video stream (Amangeldy et al., 2023b; Wang et al., 2025). The evolution of these computational approaches is summarized in Figure 1.

**Figure 1:**
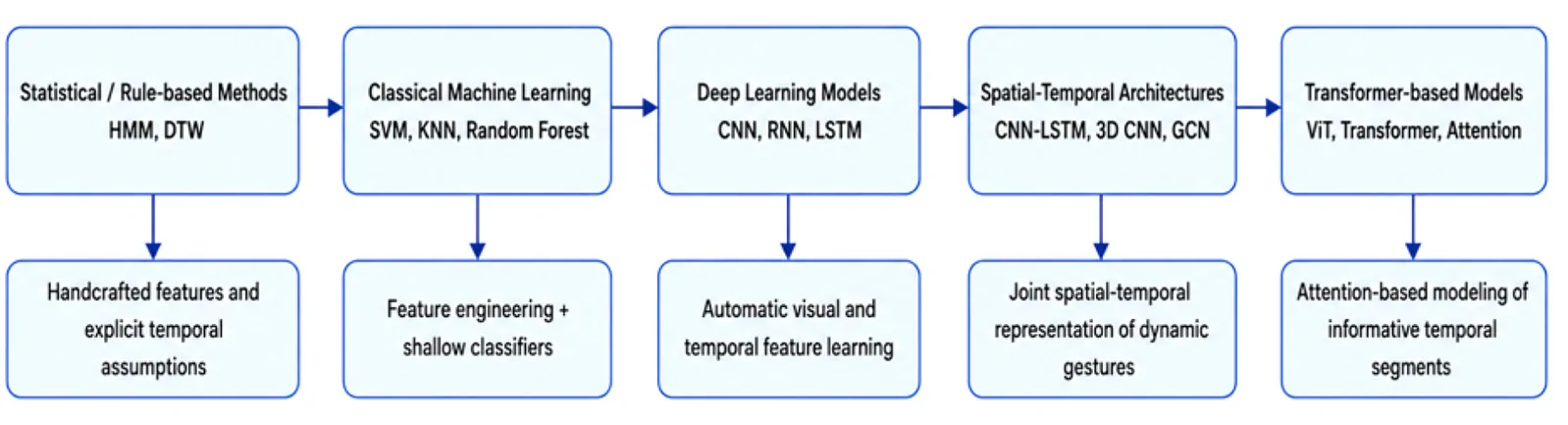
A journey from statistical approaches, rule-based approaches, toward Transformer-based approaches in sign language recognition.

While significant progress has been made in deep learning architectures, continuous sign language recognition (CSLR) is still one of the most challenging tasks, where the system is required to recognize a continuous flow of sign language gestures without clear separation between them and also consider the impact of coarticulation (Khan et al., 2025). The theoretical basis of modern sign language translation systems has focused on the importance of not only manual features (hand movements), but also non-manual features: facial expression, articulation, and body posture (Al Abdullah et al., 2024). While hybrid CNN-RNN models can be used, hybrid CNN-Transformer models have been shown to achieve even better results when employing such features, particularly for handling complex linguistic structures (Al Abdullah et al., 2024; Khan et al., 2025).

In this aspect, contemporary SLR systems increasingly adopt methods for obtaining skeletal and keypoint-based representations, one of which is the MediaPipe Holistic framework (Al Abdullah et al., 2024) that is widely used. In parallel, there is also a growing interest in real-time recognition systems that try to achieve robustness to lighting variations and minimize response time to milliseconds (Cui et al., 2025). Despite this technological advancement, there is a serious problem between developers and end users (Lepp et al., 2025). The co-creation concept sees sign language not as a “dataset,” but as a “language as people,” and demands that native signers take part in all aspects of a project, from conception to implementation. The only way that systems can be developed which consider cultural sensitivities and actual needs of the deaf community is with such an ethical and participatory approach (Lepp et al., 2025).

To date, the most dynamic use of SLR technologies is in the area of interactive mobile applications for social communication enhancement of deaf and hard-of-hearing individuals (Syafira and Yuana, 2026). In education and healthcare, AI-powered real-time translation, gesture recognition, and speech-to-text capabilities foster social integration and enhance user autonomy. Besides, mobile apps are increasingly being adopted as tools to teach users sign language via gamification and interactive sign language videos. Syafira and Yuana (2026) have conducted a systematic review of 21 articles (2019–2025) and found that 3D gesture animation, virtual avatars, and ambient sound notification systems are the most popular features when making everyday communication more accessible.

Despite the rapid advancement of research, the allocation of linguistic and computational resources in the area of SLR is still very uneven. A recent report shows that there are 119 sign languages all over the world, but only a small number of these sign languages have linguistic resources and annotated data adequate for the development of effective SLR systems (Martínez-Sánchez et al., 2023; Sultan et al., 2022). Of these, approximately 38 languages have organized lexicons and over 1,000 glosses are annotated. Research on high-resource sign languages continues to focus on American Sign Language (ASL), British Sign Language (BSL) (Al Abdullah et al., 2024; Sami et al., 2026) and German Sign Language (DGS). The existing RWTH-PHOENIX (Martínez-Sánchez et al., 2023) and ASLLVD (Tang et al., 2025) datasets are the main standards for SLR and generation tasks, respectively. Currently, the two main standards are the RWTH-PHOENIX dataset for SLR and the ASLLVD dataset for sign language generation (Martínez-Sánchez et al., 2023; Tang et al., 2025). By comparison, there are very few resources with annotations in KzSL, and no public benchmark to which it could be compared, making KzSL a low-resource sign language.

With the limitations that have already been identified for existing SLR systems, such as using large datasets, high variation of gestures between signers, and real-time operation, this study proposes a compact deep learning architecture that is aimed at recognizing KzSL in a limited data setting. In contrast to high-resource solutions which are trained on thousands of classes and hundreds of signers, the data set of this study consists of 187 output classes (186 gesture classes and one no_event class) recorded by five signers. The data collection protocol was designed to collect multiple renditions of each gesture for a number of available signers, usually 20-25 per gesture, but some gestures have uneven signer coverage in the final corpus due to the unavailability of signers. This is a challenging setting because the model needs to learn stable spatiotemporal features from the small amount of data with low balance, and needs to generalize to signers not seen during the training process.

In order to solve this issue, a lightweight multi-branch Transformer is proposed, which is based on the landmarks from the MediaPipe Holistic model. The gesture sample is a fixed temporal landmark tensor of 56 × 1,662, which represents the body, hands, and face. Instead of a homogeneous vector that represents the frame in the 1,662-dimensional input, the architecture uses separate projection branches for pose, face, left-hand, and right-hand landmarks, indicating their different roles in sign language. A Transformer encoder is then used to temporally model the fused representation, where the self-attention mechanism in the Transformer is able to capture short-range and long-range dependencies between the different phases of the gesture, while a temporal attention pooling module highlights the most informative parts of the gesture. The proposed model falls somewhere between the heavy video-based Transformer and GCN models and classic lightweight ones: it can model the temporal structure of a gesture while remaining comparatively compact, though its suitability for real-time deployment on SLR systems and social digital platforms remains to be established through dedicated, identical-hardware latency benchmarking (Section 4).

This study has the following contributions. The five signers provided studio-quality RGB video, and a KzSL dataset was created using MediaPipe Holistic landmark extraction with 187 output classes containing 39,979 gesture samples. Based on this, a lightweight multi-branch Transformer model (SignTransformerModel) which processes the pose, face, left-hand shape, and right-hand shape landmarks with different branches, respectively, followed by temporal self-attention modeling to capture temporal information, retaining body-part-specific information of body parts in the manual and non-manual parts of sign language, was proposed and developed for landmark-based KzSL recognition. The model was evaluated with a nested, signer-independent Leave-One-Signer-Out cross-validation protocol, in which the training duration for each held-out partition is selected without reference to that partition, to obtain a realistic and leakage-free estimate of the generalization performance to unseen signers; the proposed model was further compared against three baseline architectures—a Standard Transformer, a Temporal Convolutional Network, and a Bidirectional GRU—trained and evaluated under an identical protocol, to establish whether the multi-branch design offers a measurable advantage over simpler alternatives. A class-level error analysis was performed to detect common confusions and structural limitations of the dataset that prevent achieving high recognition.

## 2 Materials and methods

This study adopted the experimental design of isolated sign language recognition. The whole pipeline involves four stages: acquiring RGB video of the KzSL gestures in a controlled studio setting; extracting pose and face landmarks with MediaPipe Holistic and mapping them to standardized temporal tensors, and extracting hand landmarks and mapping them to standardized temporal tensors; training the proposed multi-branch Transformer model together with three baseline architectures for comparison; and signer-independent evaluation through a nested Leave-One-Signer-Out cross-validation, with class-level and confusion analysis. The recognition task is defined as multi-class classification of a landmark sequence into one of 187 output classes (186 gesture classes plus one class of no event).

### 2.1 Dataset and data acquisition

For this research, a special dynamic set of Kazakh Sign Language gestures was developed. Collected using a specially prepared studio environment and a vision-based approach with RGB video recording. A green screen, professional lighting equipment, and GoPro HERO 12 digital video cameras with a video resolution of 3,840 × 2,160 (4K) were used during recording, which maintained consistent image quality while reducing the effects of background noise and illumination variations. The recording environment is shown in Figure 2.

**Figure 2:**
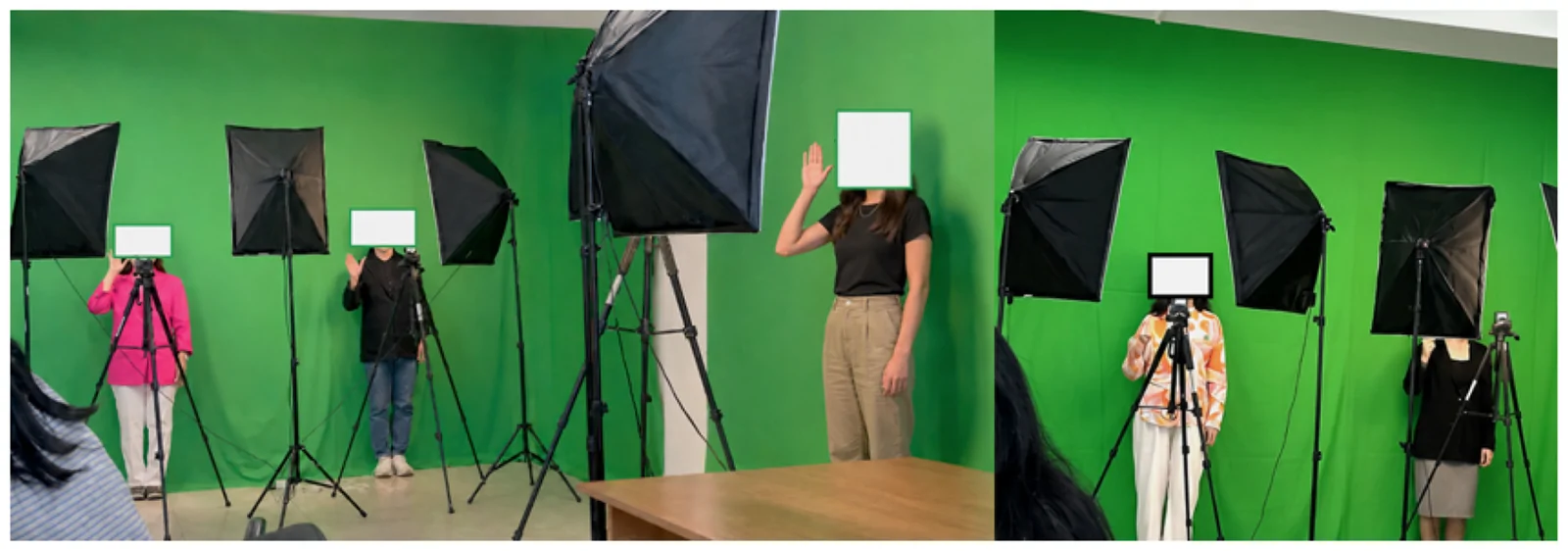
Studio environment used for Kazakh Sign Language dataset acquisition, with green-screen background, professional lighting, and fixed camera positions.

Five sign language signers (denoted as a, b, j, m, and n) participated in the creation of the dataset. The recordings were designed to capture repeated performances of each gesture by multiple signers in order to reflect intra- and inter-signer variability and to enhance the model's robustness to variations in individual signing styles. On average, each gesture was recorded 20–25 times by the available signers; however, the final corpus contained uneven signer coverage for several classes, which was taken into account during the signer-independent evaluation protocol. An example of repeated recordings is presented in Figure 3.

**Figure 3:**
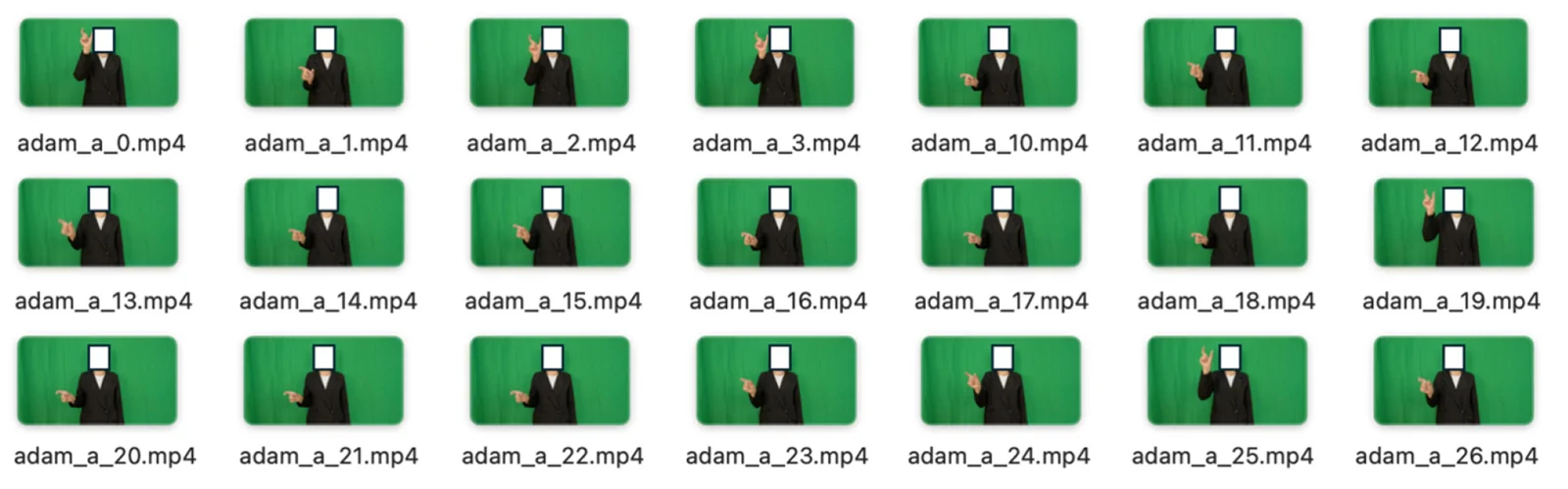
Example of repeated recordings of a single gesture class performed by one signer, illustrating intra-signer variability captured in the dataset.

The final corpus used for model training contained 39,979 gesture samples distributed over 187 output classes. Of these, 38,043 samples were available in the original annotation, and a further 1,936 samples were added after completing the annotation of 1,460 previously unprocessed video files. Each sample corresponds to an RGB video clip containing spatiotemporal information about hand movements, body posture, and non-manual components of sign language. A summary of the dataset and its pre-processing configuration is provided in Table 1.

**Table 1:** Brief summary of the dataset and its pre-processing.

Characteristic	Value
Sign language	KzSL
Signers	5 (a, b, j, m, n)
Total samples	39,979 (38,043 original + 1,936 after annotation of 1,460 previously missing files)
Output classes	187 (186 gesture classes + 1 no_event class)
Recording setup	RGB video, green-screen studio, GoPro HERO 12 (3,840 × 2,160)
Landmark extraction	MediaPipe Holistic
Features per frame	1662 (pose 33 × 4; face 468 × 3; left hand 21 × 3; right hand 21 × 3)
Temporal length	Resampled to T = 56 frames
Normalization	Per-sequence z-score
Augmentation	Frame dropout (*p* = 0.1); Gaussian noise (σ = 0.003)

### 2.2 Landmark extraction and pre-processing

Hand, body and face landmarks were extracted from the frames of the video using MediaPipe Holistic for further analysis. Each frame was then described as a 1,662-dimensional feature vector, where the 33 pose landmarks are described by four values (x, y, z, visibility) and the 468 face landmarks, the 21 left-hand landmarks, and 21 right-hand landmarks are all described by three.

The data was then pre-processed after landmark extraction to ensure stability of the models and to ensure uniform input sequences. The first step was to automatically segment the video stream for the identification of the active phase of each gesture. For this purpose, a motion score was computed from the pose and both hand landmark blocks only (face landmarks were excluded from this calculation); for each pair of consecutive frames, the Euclidean distance between their concatenated pose-and-hand feature vectors was computed, and the resulting sequence was smoothed with a length-9 moving average. An activity threshold was set separately for each recording as threshold = median (motion) + 3 × [MAD (motion) + 10^−6^], where MAD denotes the median absolute deviation; frames with a motion score above this threshold were classified as active. Active frames separated by a gap of at most two indices were merged into a single interval, and, when more than one candidate interval remained, the longest was selected as the gesture segment; its boundaries were then extended by two frames before the start and four frames after the end (clipped to the recording length) to avoid truncating the gesture. If fewer than three frames exceeded the threshold, the central 20–80% of the recording was retained instead, and recordings shorter than 10 frames were kept in full; segments shorter than two frames were discarded, and no explicit maximum segment duration was enforced at this stage. Sufficiently long intervals immediately preceding and following the selected gesture segment (at least 28 frames each) were retained and labeled as no_event; because this labeling rule is applied automatically, it does not guarantee that every no_event segment is entirely free of gesture-related motion. All frames that were not informative, that is, those outside the selected gesture and no_event intervals, were discarded during this process. In addition to this automatic segmentation, all trimmed video recordings were subsequently reviewed manually to check the correctness of the resulting gesture boundaries; quality control therefore combined this visual inspection with the automated validation of the feature arrays described below. The landmark extraction and pre-processing pipeline is illustrated in Figure 4.

**Figure 4:**
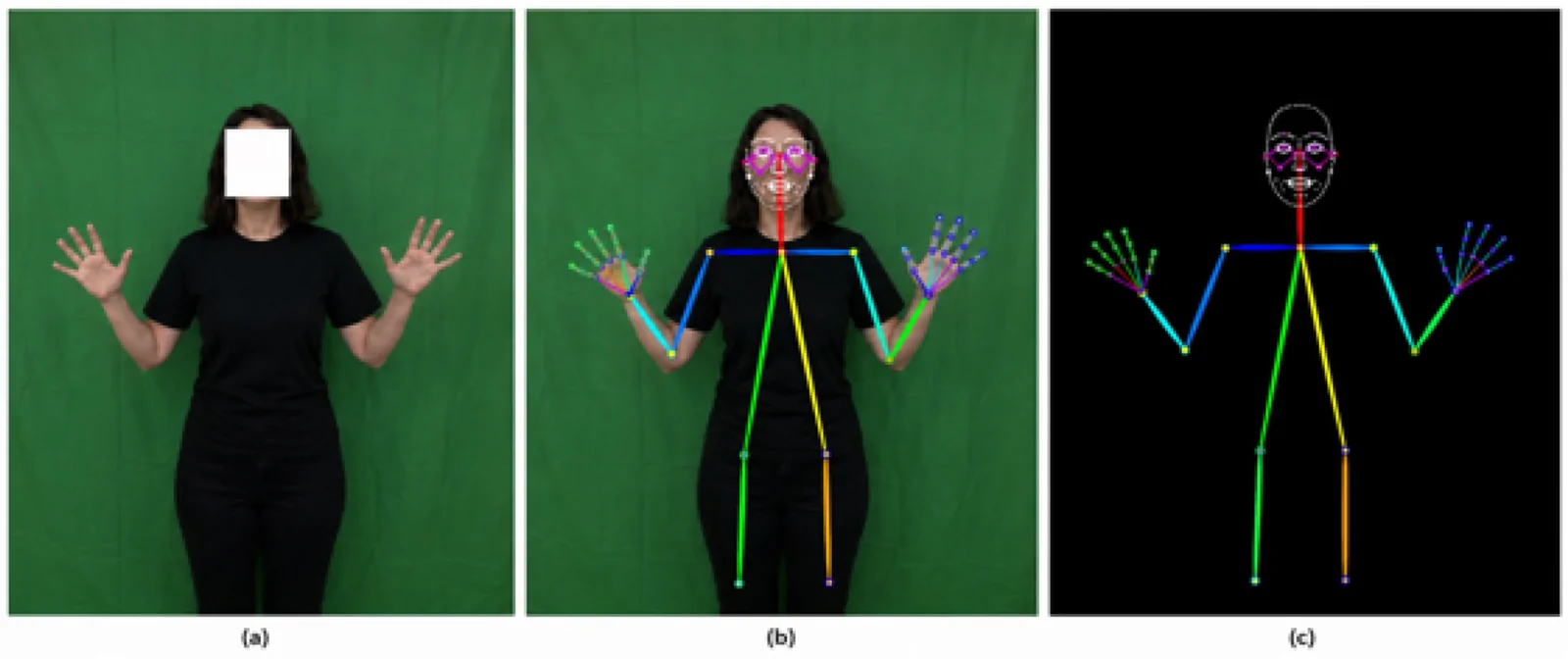
Pipeline of landmark extraction for KzSL recognition: **(a)** original RGB frame; **(b)** MediaPipe Holistic landmarks overlaid on the frame; **(c)** the same landmarks shown in isolation, with the video background removed for visualization.

When MediaPipe Holistic failed to detect a hand, face, or pose in a given frame, the corresponding feature block was set to zero rather than being reconstructed from neighboring frames; no explicit missing-data mask, separate instability filter, or dedicated interpolation across missing detections was applied beyond this zero-filling, and the subsequent resampling to 56 frames treats these zero-valued frames the same as any other frame rather than as a special case.

To improve the model's generalization performance, a few data augmentation techniques are used. The landmark coordinates were corrupted by Gaussian noise with a standard deviation of 0.003 to make the model less susceptible to overfitting on the exact landmark position and more robust to realistic conditions of video capture where visual interference and input variation occur, while frames were dropped with probability *p* = 0.1 to simulate missing frames and enhance the robustness of the model with respect to temporal instability in the video stream. Both augmentations were applied only to the training split within each fold, never to validation or test data.

The entire set of sequences was then brought to a fixed length of T = 56 frames using linear temporal interpolation, which standardized the input data irrespective of differences in the speed and duration of gestures performed by different signers. After temporal normalization, z-score normalization of the features was applied independently within each clip: for each feature, the mean and standard deviation within that clip's own sequence were computed and used to standardize the values, so that no statistics were shared across clips or across the training and validation splits. This reduced the influence of individual differences between signers and increased the robustness of the model to variations in movement amplitude. The resulting standardized tensor representation is shown in Figure 5. All 39,979 prepared clips were validated to confirm a uniform shape of 56 × 1,662 with no NaN or infinite values, no all-zero clips, and no exact duplicate arrays.

**Figure 5:**
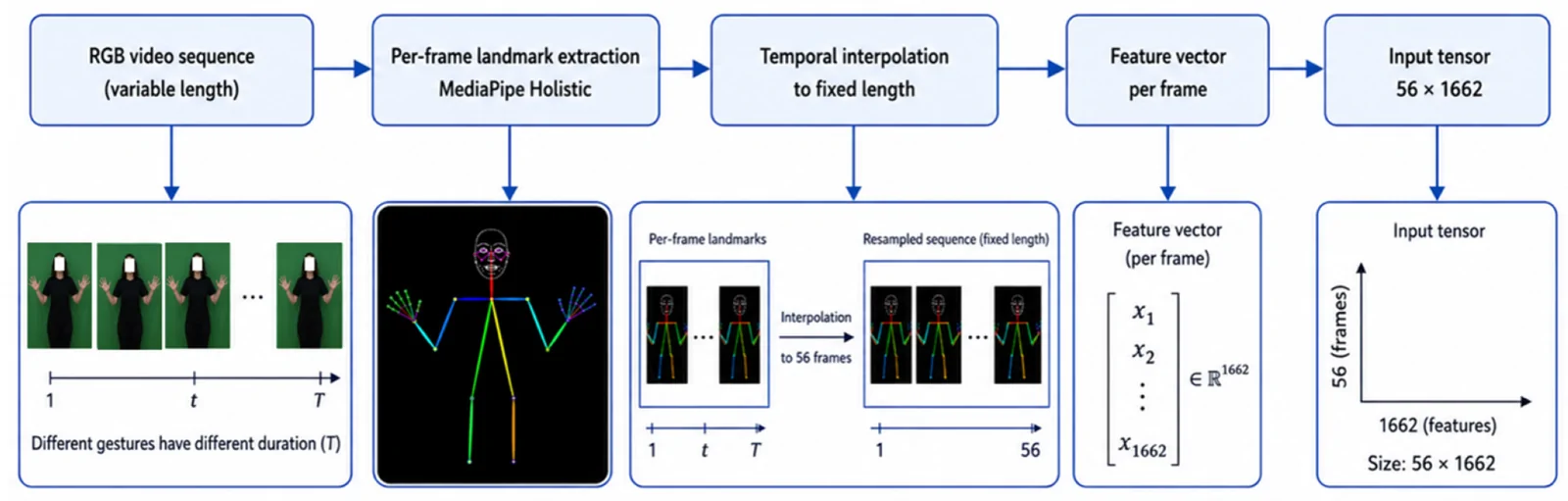
Transformation of temporal landmark sequences into a standardized input tensor of size 56 × 1,662 for deep learning-based gesture recognition.

The resulting dataset reflects the specific characteristics of Kazakh Sign Language and captures realistic variation in gesture execution by different native signers, which is particularly important given the very limited number of publicly available KzSL datasets.

### 2.3 Proposed multi-branch transformer model

The recognition model developed in this study, SignTransformerModel, is a lightweight multi-branch Transformer designed to process temporal sequences of sign language landmark features. The architecture consists of five sequential modules—multi-branch input stem (MultiBranchStem), sinusoidal positional encoding, a stack of Transformer encoder layers, temporal attention pooling module (TemporalAttentionPool), and classification head—and has around 3,529,272 trainable parameters, making it a compact architecture for possible real-time deployment. The overall structure is shown in Figure 6.

**Figure 6:**
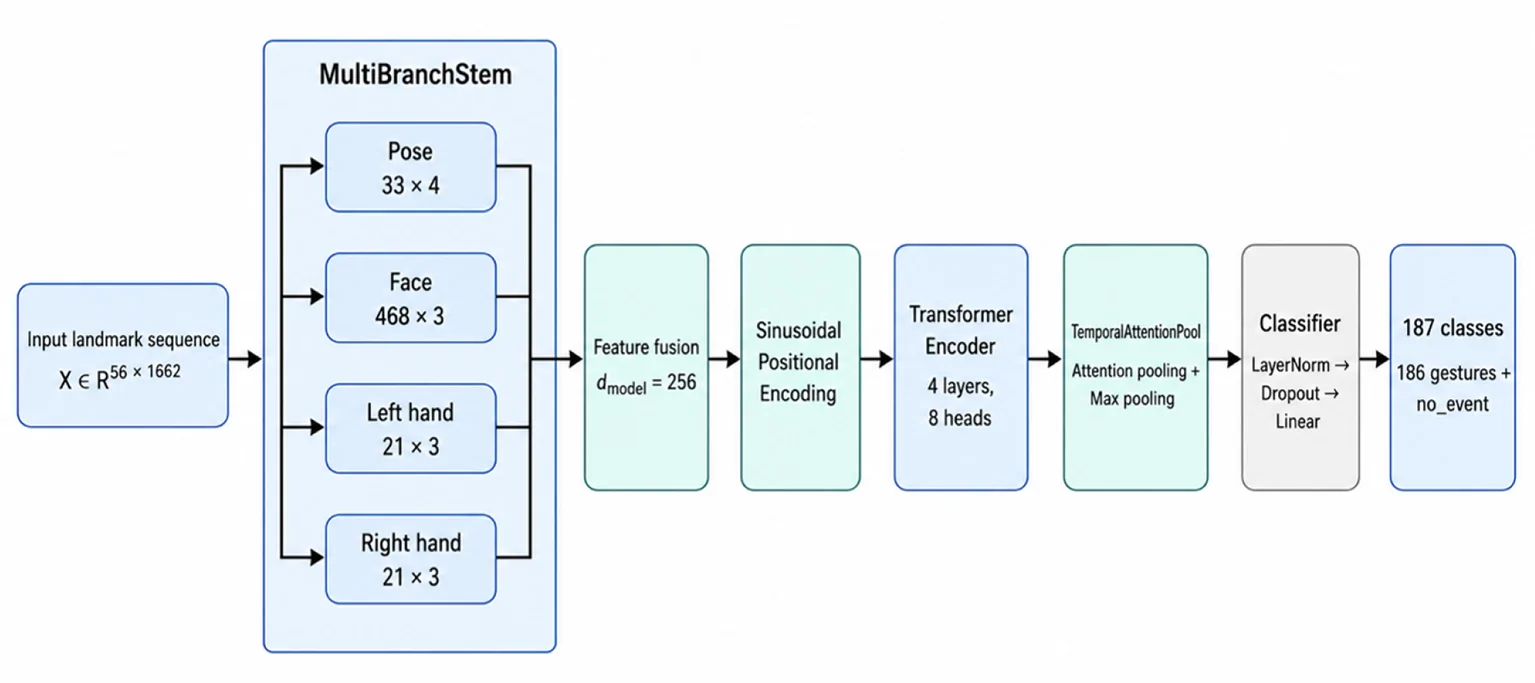
The proposed multi-branch transformer architecture for Kazakh Sign Language recognition.

Every gesture sample is a fixed temporal landmark tensor X, a 56 × 1,662 matrix, where 56 corresponds to the number of time steps after applying temporal interpolation, and 1,662 corresponds to the number of MediaPipe Holistic features extracted from each time step. The per-frame vector concatenates four anatomically distinct groups of features: pose landmarks (33 × 4 = 132 values), face landmarks (468 × 3 = 1404 values), left-hand landmarks (21 × 3 = 63 values), and right-hand landmarks (21 × 3 = 63 values).

Instead of projecting the entire 1,662-dimensional frame vector into one linear layer, the MultiBranchStem splits up the input into its anatomical parts: pose, face, left hand, and right hand, each of which is projected separately, namely the pose branch to d_model/4 dimensions, the face branch to d_model/4 dimensions, and each hand branch to d_model/8 dimensions, where d_model = 256. The outputs of the branches are then combined into a single representation, d_model-dimensional, for every time step. This design reflects the linguistic structure of sign language. Pose, face, and hands carry different semantic roles: the hands convey the primary manual articulation of a sign, the body posture provides spatial reference and movement context, and the face contributes non-manual markers such as facial expression and articulation. Processing these components through separate projections allows the model to preserve body-part-specific information before temporal modeling, prevents the numerically dominant face features (1404 of 1662 dimensions) from overwhelming the comparatively low-dimensional but highly discriminative hand features, and supports the joint modeling of manual and non-manual components that prior work has identified as critical for accurate SLR (Al Abdullah et al., 2024; Khan et al., 2025).

Because the self-attention operation in a Transformer is permutation-invariant, the encoder has no intrinsic notion of temporal order. To preserve the ordering of gesture phases, sinusoidal positional encodings (with a maximum supported length of 256 positions) are added to the fused frame representations before they enter the encoder, injecting information about the absolute position of each frame within the sequence and enabling the model to distinguish, for example, the preparation, stroke, and retraction phases of a gesture.

The temporal core of the model is a stack of four TransformerEncoderLayer blocks, each with eight attention heads, a feed-forward expansion multiplier of 4, and GELU activation. Multi-head self-attention lets the model “attend to all the other time steps,” enabling it to learn long-range temporal dependencies between the different phases of a gesture—such as how the first hand configuration relates to the final position for the gesture—without the sequential limitations of recurrent architectures. The multiple heads allow the model to simultaneously process various aspects of the movement, including changes in posture, hand trajectory and facial dynamics.

The TemporalAttentionPool module merges together 56 frame representations into a single vector by combining two complementary mechanisms: attention pooling, which learns to pay more attention to the most informative temporal regions of the gesture, and max pooling, which retains the highest discriminatory activations through the gesture. The outputs of both the pooling paths are combined into a fixed-size representation, enabling the model to focus adaptively on the segments that contain the salient information for the sign, while still preserving salient high-activity outputs that could be muddled by averaging. Finally, the fused representation is fed into a smaller classification head that includes layer normalization, dropout, and a linear layer that maps the d_model-dimensional vector to 187 output logits [LayerNorm → Dropout → Linear(d_model, 187)], followed by a softmax function to generate a probability distribution over the 187 output classes.

This study asks whether the performance of SignTransformerModel is attributable specifically to the multi-branch input design and the Transformer-based temporal core, rather than to the choice of landmark representation or to overall model capacity. To answer this, three additional architectures were implemented and evaluated under an identical data pipeline, training protocol, and evaluation procedure (see the following subsection). All baselines receive the same 56 × 1,662 landmark tensor described in Section 2.2 and are trained with the same optimizer, learning-rate schedule, loss function, class weighting, and data augmentation as SignTransformerModel.

The Standard (single-stem) Transformer isolates the contribution of the MultiBranchStem. Instead of splitting the 1,662-dimensional per-frame vector into separate pose, face, left-hand, and right-hand branches, a single linear layer projects the full frame vector into a d_model = 256-dimensional representation. The resulting sequence is passed through the same sinusoidal positional encoding, the same four-layer Transformer encoder (eight attention heads, feed-forward multiplier 4, GELU activation), the same TemporalAttentionPool module, and the same classification head as SignTransformerModel. Because the temporal core and the pooling mechanism are kept fixed, any performance difference between this baseline and the proposed model can be attributed to the anatomical branch separation itself rather than to the self-attention mechanism; this architecture has 3,802,552 trainable parameters.

The Temporal Convolutional Network follows the general design of temporal convolutional networks used for sequence modeling. A stack of dilated one-dimensional convolutional blocks with residual connections is applied along the temporal axis of the 56 × 1,662 input. Each block combines a dilated convolution, weight normalization, GELU activation, and dropout, with dilation increasing exponentially across blocks so that the receptive field spans the full 56-frame window without recurrence. The resulting sequence is aggregated with the same TemporalAttentionPool module and classification head used in the other architectures, so that the comparison isolates the effect of the temporal backbone (convolutional vs. self-attention) rather than the pooling or classification stage; this architecture has 1,691,580 trainable parameters.

The Bidirectional GRU (BiGRU) baseline is a stacked bidirectional Gated Recurrent Unit network that processes the landmark sequence frame by frame in both temporal directions, providing a recurrent baseline representative of the LSTM/GRU family discussed in the Section 1 (Rastgoo et al., 2024; Bahuguna et al., 2025). As with the other baselines, the final hidden representations are aggregated with the TemporalAttentionPool module and passed to the same classification head; this architecture has 1,236,408 trainable parameters.

### 2.4 Training and evaluation protocol

The AdamW optimizer with a learning rate of 3 × 10^−4^ and weight decay of 10^−3^ was used to train the model, and a cosine annealing learning rate schedule (CosineAnnealingLR, T_max = 125) was adopted. This schedule was retained, with the same horizon, in both the inner cross-validation runs and the final refitting runs described below. For training, mini-batches of 128 were used. The model was optimized using the cross-entropy loss function with a label smoothing factor of 0.08, and class weights were used based on the square root of the inverse class frequency (sqrt_inv) to reduce the class imbalance. To enhance the stability of the optimization process, gradient clipping with max_norm = 1.0 was applied; during training, data augmentation included frame dropout (p = 0.1) and additive Gaussian noise to the landmark coordinates (σ = 0.003), as described in Section 2.2. All experiments used a single fixed random seed (seed = 42). The complete training configuration is given in Table 2. All experiments were run on Python 3.11.11 (Python Software Foundation, Beaverton, Oregon, USA) with NumPy 2.2.1 and PyTorch 2.7.0 (PyTorch Foundation (Linux Foundation), Wilmington, Delaware, USA) (CUDA runtime 12.6) on NVIDIA Quadro RTX 6000 GPUs.

**Table 2:** Training hyperparameters.

Parameter	Value
Model	SignTransformerModel (multi-branch Transformer)
Input size	56 × 1662
d_model	256
Transformer encoder layers	4
Attention heads	8
Feed-forward multiplier	4
Activation	GELU
Trainable parameters	3,529,272
Epochs (inner cross-validation runs)	125 (fixed; no early stopping)
Refitting duration (final model)	Selected per outer test partition via inner cross-validation; range 101–119 epochs across the five partitions (see Training and Evaluation Protocol)
Batch size	128
Optimizer	AdamW
Learning rate	3 × 10^−4^
Weight decay	10^−3^
Scheduler	CosineAnnealingLR (T_max = 125)
Loss function	CrossEntropyLoss, label smoothing = 0.08
Class weights	sqrt_inv (square root of inverse class frequency)
Augmentation	frame_dropout_p = 0.1; noise_std = 0.003
Gradient clipping	1.0

To eliminate a data-leakage concern—under the original protocol, the held-out signer was used both to select the number of training epochs, via early stopping on macro F1, and to report the final performance for that same signer—a nested cross-validation design was adopted in place of a single-level Leave-One-Signer-Out (LOSO) evaluation. The dataset was partitioned into the same five signer-labeled groups described in Section 2.1 (a, b, j, m, and n).

For each of the five partitions in turn, that partition was designated the outer test partition and withheld entirely from training and from model selection. The remaining four partitions underwent an inner leave-one-partition-out cross-validation: for each of the four possible choices of inner validation partition, a model was trained from a random initialization on the other three partitions and evaluated, epoch by epoch, on the inner validation partition, for the full 125-epoch budget, without early stopping. The four resulting per-epoch macro F1 curves were averaged, and the epoch attaining the highest mean macro F1 across the four inner validation partitions was selected as the refitting duration for that outer test partition. A new model, again from a random initialization, was then trained on all four development partitions combined for the selected number of epochs, with no validation split during this refitting stage, and evaluated exactly once on the outer test partition that had been withheld from the start. This procedure was repeated independently for each of the five outer test partitions, so that no sample from an outer test partition, and no metric computed on it, were used to select the epoch count or any other configuration choice for that partition before its single, final evaluation.

The final signer-independent evaluation was performed on the five outer test partitions corresponding to the signer-labeled groups: a, b, j, m, and n, each evaluated once after a refitting run whose duration was selected without reference to that partition. The training set for each partition's refitting run consisted of data from the other four partitions, so that the evaluation is carried out in a condition of signer independence. All reported fold-level, class-level, and confusion-based results were obtained using this nested protocol; results obtained under the original, single-level LOSO protocol are reported separately, for comparison, in the Discussion.

Three metrics were used for model performance: overall accuracy, macro-averaged F1 score (which assigns equal weights to all classes and is thus sensitive to rare and hard classes), and weighted F1 score (which averages per class F1 score according to class support). Macro F1, averaged across the four inner validation partitions, was used to select the refitting duration for each outer test partition, rather than as an early-stopping criterion within a single training run. Per-class precision, recall and F1 were also calculated and aggregated over the five outer test partitions, and a confusion analysis was carried out to highlight the gesture pairs that are systematically confused.

## 3 Results

### 3.1 Cross-validation performance

The results for the nested cross-validation are summarized in Table 3. Across all five outer test partitions, the model achieved a mean accuracy of 0.6698 ± 0.0376, a mean macro F1 of 0.4207 ± 0.0420, and a mean weighted F1 of 0.6546 ± 0.0375, outperforming the three baseline architectures compared in this study (Table 4).

**Table 3:** Nested cross-validation results: proposed model vs. baseline architectures (mean ± SD across five outer test partitions).

Architecture	Trainable parameters	Accuracy	Macro F1	Weighted F1
Multi-branch transformer (proposed)	3,529,272	0.6698 ± 0.0376	0.4207 ± 0.0420	0.6546 ± 0.0375
TCN	1,691,580	0.6588 ± 0.0374	0.3957 ± 0.0408	0.6446 ± 0.0369
Standard transformer	3,802,552	0.6216 ± 0.0457	0.3469 ± 0.0415	0.6073 ± 0.0423
BiGRU	1,236,408	0.6019 ± 0.0539	0.3163 ± 0.0509	0.5853 ± 0.0488

**Table 4:** Macro F1 by outer test partition, across architectures, with development (training) and outer test sample counts.

Partition	Train samples	Test samples	Multi-branch	TCN	Standard	BiGRU	Multi −TCN
a	32,240	7,739	0.4897	0.4658	0.4136	0.3890	+0.0239
b	32,989	6,990	0.4262	0.4113	0.3610	0.3360	+0.0149
j	31,974	8,005	0.3822	0.3643	0.2983	0.2418	+0.0179
m	31,274	8,705	0.4333	0.3868	0.3540	0.3353	+0.0465
n	31,439	8,540	0.3719	0.3500	0.3076	0.2792	+0.0219

The highest macro F1 (0.4897) was achieved by partition a, showing that the gestures of this partition were relatively well-predicted by the remaining partitions' styles. The lowest macro F1 (0.3719) was found on partition n. Table 5 reports the corresponding per-partition accuracy and weighted F1 for the proposed model: partition a also attained the highest accuracy (0.7068) and weighted F1 (0.6888), while partition j attained the lowest accuracy (0.6034) and weighted F1 (0.5891)—a ranking that differs from the macro F1 ranking above, reflecting the difference between class-balanced and support-weighted evaluation. Under the original, single-level LOSO protocol, signer j had been identified as the weakest fold and reported as not fully converged within its epoch budget; under the corrected nested protocol, j remains the weakest partition by accuracy and weighted F1, though partition n is now weakest by macro F1, and no equivalent convergence issue has been established for either partition. This shift between protocols is discussed further in Section 4.

**Table 5:** Accuracy, macro F1, and weighted F1 by outer test partition, proposed multi-branch Transformer.

Partition	Accuracy	Macro F1	Weighted F1
a	0.7068	0.4897	0.6888
b	0.6960	0.4262	0.6882
j	0.6034	0.3822	0.5891
m	0.6886	0.4333	0.6684
n	0.6541	0.3719	0.6387

The reported results are obtained from five separate refitting runs, each trained on the four development partitions corresponding to that outer test partition, rather than a single configuration trained on the full set of 39,979 samples. These runs are the focus of the below analysis, and the results are reported as partition-level performance, class-level recognition quality, and the most common confusion patterns.

The partition-level results demonstrate performance that varies with the outer test partition, but at the same time highlight that there is a difference between the best and worst partitions, which reflects the inter-signer variability. The relatively low macro F1 further indicates a difference with the weighted F1, suggesting there is a difference between class imbalance and class difficulty in recognizing quality, which is explored further in the next subsection.

### 3.2 Class-level performance and error analysis

The no_event class accounts for 16,794 of the 39,979 samples in the corpus (42.01%) and is recognized with near-ceiling F1 by every architecture tested (Table 6), so the aggregate accuracy and weighted F1 reported in Table 3 are substantially influenced by this single, dominant, non-gestural class. Restricting accuracy to samples whose true label is a gesture (a prediction of no_event for such a sample still counts as an error) reduces the proposed model's accuracy from 0.6698 to 0.4490—a reduction of more than 22 percentage points—indicating that the aggregate accuracy in Table 3 should not be read as a measure of gesture-recognition accuracy in isolation. Table 6 reports this gesture-only accuracy, macro F1 computed over the 186 gesture classes, and the mean per-model F1 on no_event itself, for all four architectures; the ranking is unchanged from Table 3, with the multi-branch Transformer remaining best, followed by TCN, the Standard Transformer, and BiGRU.

**Table 6:** Performance excluding the no_event class, by architecture.

Architecture	Accuracy on gesture samples	Macro F1 (186 gesture classes)	Mean F1, no_event
Multi-branch transformer (proposed)	0.4490 ± 0.0471	0.4178 ± 0.0422	0.9578
TCN	0.4266 ± 0.0510	0.3926 ± 0.0410	0.9714
Standard transformer	0.3714 ± 0.0514	0.3436 ± 0.0417	0.9531
BiGRU	0.3414 ± 0.0573	0.3129 ± 0.0511	0.9445

Under the original, single-level LOSO evaluation, per-class analysis revealed substantial heterogeneity behind the aggregate metrics: 59 of 187 classes (approximately 32%) reached a mean F1 of at least 0.5, whereas 128 classes (approximately 68%) remained below this threshold; ten classes reached F1 ≥ 0.7 and four classes reached F1 ≥ 0.8. These corpus-wide class-level statistics have not yet been recomputed under the nested protocol; the best- and worst-recognized classes are summarized in Table 7.

**Table 7:** Best- and worst-recognized classes.

Group	Class	Precision	Recall	F1	Support
Best	no_event	0.955	0.963	0.9578	3359
Best	Tusinu	0.841	0.912	0.871	24.6
Best	Eger	0.822	0.843	0.826	25.2
Best	Qatty_gromkii	0.791	0.828	0.805	23.4
Best	Qar	0.748	0.824	0.762	25.0
Worst	Mumkin	–	–	0.0000	65
Worst	Ozgertu	–	–	0.0168	140
Worst	Kutu	–	–	0.0581	121
Worst	Qalai	–	–	0.0667	122
Worst	Nege	–	–	0.0698	129

F1 for no_event is from the nested protocol; precision, recall, and F1 for the other best-recognized classes are from the original single-level LOSO evaluation. Support for the best-recognized classes is the mean per outer test partition; support for the worst-recognized classes is the total across the corpus.

The no_event class, which received the largest support, continues to be recognized with the highest F1 score under the nested protocol (0.9578), showing the model was very effective in distinguishing target gesture videos from videos without a target gesture. The highest F1 under the original evaluation (between 0.76 and 0.87) was obtained in the gestures Tusinu, Eger, Qatty_gromkii and Qar among the gesture classes; these best-recognized classes have not yet been re-audited under the nested protocol. The class Mumkin earned an F1 of 0.000, which is the lowest, and did so consistently under all four architectures evaluated in this study, not only under the proposed multi-branch Transformer. This result is primarily explained by incomplete signer coverage: Mumkin was recorded only by signers b, j and m, and is entirely absent from signers a and n, so that it is evaluated in only three of the five outer test partitions and, in each of those, the model is trained on this class using data from only the other two of the three contributing signers. This gesture does not therefore have a model that learns a representation that is signer-invariant enough. This inability isn't, therefore, architectural, but rather it is a symptom of limited signer coverage—a diagnosis strengthened by its consistency across all four architectures compared. Other classes with low performance, such as Kutu, Qalai, Nege, and Ozgertu, remain low under the nested protocol as well; precision and recall for these classes have not yet been recomputed, so only F1 is reported here.

Table 8 extends this diagnosis from Mumkin to the full corpus: 158 of the 187 classes—157 gesture classes plus no_event—are recorded by all five signers, while 29 of the 186 gesture classes have incomplete coverage. Of these 29, three (Birge, Biz, and Mumkin) are recorded by only three signers, and the remaining 26 by four. The missing signer is not evenly distributed: 22 of these 26 four-signer classes are missing specifically from signer b, a further two (Bari, Bizde) from signer j, one (Ozgertu) from signer a, and one (Tamaq) from signer n, indicating that incomplete coverage in this corpus is concentrated in a small number of signers' availability during recording rather than distributed at random across the vocabulary. Ozgertu, one of the five lowest-performing gesture classes identified above, is among these 29 incompletely covered classes; by contrast, Kutu, Qalai, and Nege are covered by all five signers, so their low F1 cannot be attributed to incomplete signer coverage and instead points to a different, not yet investigated failure mechanism, such as visual or articulatory similarity to other classes. The complete signer-by-class sample counts for all 187 classes, including those with full coverage, are provided in the accompanying code repository (see Data Availability Statement).

**Table 8:** Classes with incomplete signer coverage (fewer than five contributing signers), full 187-class corpus.

Class	a	b	j	m	n	Total	Signers
Birge	0	0	25	25	25	75	3
Biz	31	0	0	25	31	87	3
Mumkin	0	20	21	24	0	65	3
Alys	24	0	47	25	25	121	4
Anda_sanda	25	0	55	25	25	130	4
Arqashan	26	0	49	22	25	122	4
Az	25	0	48	25	25	123	4
Azyq_tulik	26	0	27	24	25	102	4
Baiau	22	0	51	25	25	123	4
Bari	25	25	0	27	22	99	4
Bizde	25	25	0	25	25	100	4
Dene	27	0	51	23	25	126	4
Duken	27	0	50	25	22	124	4
Esik	25	0	50	25	25	125	4
Halyq	25	0	42	25	25	117	4
Janbyr	25	0	50	25	25	125	4
Januar	25	0	50	25	25	125	4
Jaqyn	26	0	50	24	24	124	4
Kop	25	0	51	25	25	126	4
Kuntizbe	25	0	53	25	22	125	4
Minez	25	0	51	24	25	125	4
Ozgertu	0	31	51	25	33	140	4
Paidaly	25	0	52	25	25	127	4
Qarsylas	25	0	52	25	25	127	4
Qysqa	25	0	52	25	25	127	4
Saqtau	25	0	47	32	31	135	4
Tamaq	21	17	22	26	0	86	4
Uzyn	25	0	50	25	26	126	4
Zian	25	0	51	27	27	130	4

The results at the class level continue to suggest that the recognition performance is highly dependent on the percentage of signers covered in a dataset and the number of examples available for each class. Under this evaluation protocol, classes with fewer signers remain more challenging to learn, as the signer-invariant representations are less likely to be formed in the model; the cross-architecture consistency of the Mumkin failure reinforces the need for using more recordings on under-represented gesture classes to expand the dataset.

Confusion analysis under the nested protocol indicates that systematic errors remain focused on a small number of gesture pairs, with the five most frequent ones being summarized in Table 9; only these top five pairs have been re-audited to date, in one direction only (true class → predicted class).

**Table 9:** Top confusion pairs for the proposed multi-branch Transformer, nested protocol.

True class	Predicted as	Number of errors
Memleket	Ul	41
Qorqu	Turu	40
Tabigat	Aua raiy	39
Dayindau	Jol	34
Ote	Sondyqtan	31

Errors remain clustered in a small number of pairs of classes, as indicated by the confusion analysis under the nested protocol. Three of the five most frequent pairs—Memleket/Ul, Tabigat/Aua raiy, and Ote/Sondyqtan—were also among the most frequent confusions under the original evaluation; two pairs prominent under the nested protocol, Qorqu/Turu and Dayindau/Jol, were reported with a substantially lower error count or not at all under the original, single-level LOSO evaluation. The results show that there are several classes that would still benefit from the use of either more discriminative feature representations, more recordings or expert review to obtain more reliable recognition.

## 4 Discussion

The results show that the multi-branch Transformer model with landmarks is a viable and promising model structure for the recognition of isolated KzSL under data-limited conditions, a claim now supported by a comparison against three baseline architectures (Table 3). Some of these design choices seem to lead to this result. The MultiBranchStem first splits the input anatomical parts, thereby keeping body-part-specific information intact and preventing the high-dimensional face information from overwhelming the comparatively low-dimensional but highly discriminative hand information; the consistent macro F1 advantage over a Standard Transformer baseline that receives the same input without this anatomical separation supports this interpretation. This design is similar to the previous work, which highlights the significance of both the manual and non-manual aspects for SLR (Al Abdullah et al., 2024; Khan et al., 2025). The Transformer encoder enables the model to capture temporal dependencies across different stages of a gesture without relying on a recurrent neural network architecture, while the TemporalAttentionPool module highlights the most informative part of the sequence and preserves salient discriminative activations by doing max pooling. This is of great value when the most informative part of a sign is contained within a relatively small temporal window (56 frames); a Temporal Convolutional Network baseline came closest to matching this performance, though still trailing the proposed model in every partition. The model has a total of 3,529,272 trainable parameters—more compact than the Standard Transformer baseline evaluated here, though not the smallest of the four architectures compared—and regularization strategies like label smoothing, weight decay, frame dropout, and coordinate noise have been applied to mitigate overfitting effects in the face of limited data. These findings at the same size of the dataset indicate that there is a significant impact on recognition performance from data completeness, class balance, and signer coverage.

It is important to emphasize the evaluation protocol in interpreting the absolute level of results. The nested cross-validation protocol is intentionally strict in that, for each partition, the validation signer is not included in the training set, and, unlike under the original LOSO protocol, no sample or metric from that partition is used to select the refitting duration either. However, preliminary experiments using the traditional random split approach (where samples from any signer can be on either side of the split) yielded significantly higher validation accuracy (around 87% Top-1), which overestimates the actual performance since this yields the model the opportunity to learn the signer-specific execution style in the training set. The results reported here are therefore a more realistic estimate of the system's performance for a new user, because they are reported in a signer-independent fashion. This spread between partitions (macro F1 between 0.3719 and 0.4897) showed that the inter-signer variability is still a significant source of error: unseen signers vary in gesture amplitude, tempo, hand dominance and their articulation idiosyncrasies, and only 5 signers were used in total, thus the model has limited potential for learning signer-invariant representations. The weakest partition (n) differs from the weakest fold identified under the original LOSO protocol (signer j, which had failed to converge completely within the epoch budget); no equivalent convergence issue has been established for partition n. The low number of signers is not unusual for low-resource sign languages like KzSL, and results showed that greater diversity of signers is likely to be the most direct way to achieve higher levels of generalization.

The significant difference in the weighted and macro F1 scores is directly caused by class imbalance and the difficulty of the classes. The no_event class accounts for 42.01% of the corpus and is recognized with near-perfect accuracy and has a high level of support, which is reflected in the support-weighted average; on the other hand, macro F1 is computed by giving equal importance to all 187 classes, and thus is depressed by rare and hard-to-recognize classes. When accuracy is restricted to gesture samples only, it falls to 0.4490, a substantially less favorable picture of gesture-recognition performance than the aggregate figure of around 2/3 weighted F1 suggests, while still having a long tail of poor class accuracy for not well-recognized classes. This tail has two failure mechanisms identified at the class level. The first form of under-representation is structural: Mumkin is signed only by signers b, j and m, and is entirely absent from signers a and n, so that under the LOSO protocol it is evaluated in only three of the five partitions and, in each, the model learns from only two of the three contributing signers when the third is held out for validation, preventing it from learning a representation that is not dependent on the signers; this diagnosis is strengthened by Mumkin obtaining an F1 of 0.000 under all four architectures compared, not only the proposed model. This suggests that the lack of signers is mostly a data problem (as opposed to a model-level problem) that needs to be solved by data collection, not just model-level modification. The second mechanism is recurrent class confusion, exemplified by Memleket/Ul, Tabigat/Aua raiy, and Ote/Sondyqtan under both evaluation protocols, and by Qorqu/Turu and Dayindau/Jol under the nested protocol as well; such errors are unlikely to be resolved by additional training epochs alone and instead require expert review of the recorded material, targeted re-recording, or features with finer spatial discrimination.

The present study is subject to several limitations that should be considered. The dataset, although purpose-built and comparatively large for KzSL, remains limited in scale and signer diversity compared with high-resource benchmarks such as RWTH-PHOENIX or ASLLVD, and five signers cannot capture the full variability of the signing population. Several classes are unevenly represented across signers, of which Mumkin is the extreme case, a failure confirmed across all four architectures compared. Rather than processing raw RGB video, the model relies on landmarks extracted using MediaPipe Holistic; while this improves compactness and invariance to background, clothing, and camera specifics, it also makes recognition quality dependent on the accuracy of the upstream landmark extractor, and appearance-based cues not captured by landmarks are unavailable to the model. The reported evaluation is offline, so real-time deployment will require additional analysis of latency and robustness—including a unified benchmark across the four architectures compared, since parameter count alone does not determine inference cost—as well as usability evaluation involving deaf and hard-of-hearing participants. The baseline comparison is based on a single random seed, and the TCN baseline specifically was trained with non-deterministic cuDNN settings, unlike the other three architectures. Finally, the interpretation of systematically confused gesture pairs requires expert review by Kazakh Sign Language specialists, which has not yet been carried out.

The obtained outcomes show that despite these restrictions, it is possible to create a compact and landmark-based recognition element for Kazakh Sign Language. The proposed model is a realistic solution for future integration on digital platforms for the KzSL, on a dictionary of sign language, on education tools, and can be integrated into assistive communication systems for deaf and hard-of-hearing users because of its landmark-based input and computational efficiency; as the co-creation approach explained in the introduction (Lepp et al., 2025) suggests that the applications should be developed with the direct involvement of the sign language community.

## 5 Conclusion

This study presented a lightweight multi-branch Transformer model for KzSL recognition based on MediaPipe Holistic landmarks. Gesture samples were represented as fixed temporal tensors of size 56 × 1,662 and drawn from a purpose-collected dataset of 39,979 samples covering 187 output classes recorded by five signers. The proposed SignTransformerModel processes pose, face, and hand landmarks through separate projection branches, models temporal structure with a four-layer Transformer encoder, and aggregates the sequence with combined attention and max pooling, totaling 3,529,272 trainable parameters. Under the nested, signer-independent cross-validation, the proposed model achieved a mean accuracy of 0.6698 ± 0.0376, a mean macro F1 of 0.4207 ± 0.0420, and a mean weighted F1 of 0.6546 ± 0.0375, the highest mean values of the four architectures compared, and outperformed all three baseline architectures in macro F1 on every one of the five outer test partitions.

The obtained results show the potential of using small landmark-based Transformer architectures for a low-resource sign language, and suggest that more work is needed in order to improve results, which mainly comes from balancing the sign language data across signers, and ensuring full coverage of the sign language classes, as well as an expert-driven review of the most often confused gesture classes; aggregate accuracy in particular remains substantially inflated by the dominant no_event class, with accuracy on gesture samples alone falling to 0.4490. The proposed framework can function as the recognition component of future Kazakh Sign Language digital platforms, dictionaries, educational materials, and assistive communication systems.

## 6 Future work

Future work follows directly from the analysis. On the data side, priority should be given to recording the under-represented classes—in particular Mumkin, which obtains an F1 of 0.000 under every architecture compared—with additional signers, and to reviewing frequently confused classes such as Memleket/Ul, Qorqu/Turu, Tabigat/Aua raiy, Dayindau/Jol, and Ote/Sondyqtan together with KzSL specialists; a pre-registered ablation that removes the no_event class from training and scoring would also help characterize gesture-recognition performance in isolation. On the evaluation side, the nested cross-validation protocol should be repeated with multiple random seeds and a significance test before the advantage over the TCN baseline is asserted beyond a single-seed observation, and the correspondence between the five outer partition labels and physically distinct signers should be documented explicitly. On the modeling side, moderate capacity increases (d_model = 384 or six Transformer encoder layers) may be explored, along with augmentation strategies for frequently confused classes and signer augmentation through random keypoint scaling to simulate additional signers. Deployment-oriented evaluation—including real-time latency across all four architectures compared, resolution of the non-deterministic cuDNN configuration used for the TCN baseline, robustness to imperfect landmark extraction, and user testing—is required before the model can be embedded in practical applications.

## Data Availability

The raw data supporting the conclusions of this article will be made available by the authors, without undue reservation.
